# (1*R*,2*R*,*E*,*E*)-*N*,*N*′-Bis(4-chloro­benzyl­idene)cyclo­hexane-1,2-diamine

**DOI:** 10.1107/S1600536812000724

**Published:** 2012-01-14

**Authors:** Hamid Arvinnezhad, Khosrow Jadidi, Behrouz Notash

**Affiliations:** a Department of Chemistry, Shahid Beheshti University, G. C., Evin, Tehran 1983963113, Iran

## Abstract

The title Schiff base ligand, C_20_H_20_Cl_2_N_2_, was prepared by condensation of commercially available *p*-chloro­benzalde­hyde and (*R*,*R*)-1,2-diammonium­cyclo­hexane mono-(+)-tartrate. The cyclo­hexane ring adopts a chair conformation. The dihedral angle between the two aromatic rings is 62.52 (8)°. The crystal structure is stabilized by an inter­molecular C—H⋯Cl hydrogen bond.

## Related literature

For the crystal structures of some Schiff bases derived from cyclo­hexane-1,2-diamine, see: Fan *et al.* (2011 ▶); Glidewell *et al.* (2005 ▶); Saleh Salga *et al.* (2010 ▶). For applications of chiral Schiff base ligands, see: Da Silva *et al.* (2011 ▶); Przybylski *et al.* (2009 ▶); Gupta & Sutar (2008 ▶); Dhar & Taploo (1982 ▶); Munslow *et al.* (2001 ▶); Gillespie *et al.* (2002 ▶); Kureshy *et al.* (2001 ▶); Takenaka *et al.* (2002 ▶). For the synthesis of the title compound, see: Larrow & Jacobsen (1998 ▶); Periasamy *et al.* (2001 ▶).
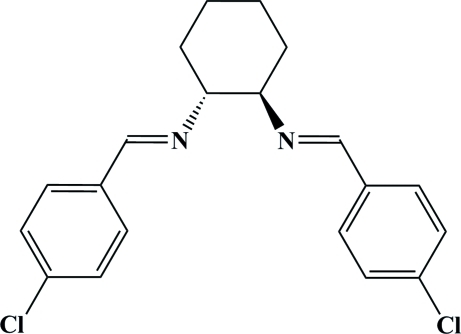


## Experimental

### 

#### Crystal data

C_20_H_20_Cl_2_N_2_
*M* *_r_* = 359.28
Orthorhombic, 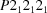

*a* = 5.5058 (11) Å
*b* = 15.734 (3) Å
*c* = 21.302 (4) Å
*V* = 1845.4 (6) Å^3^
*Z* = 4
Mo *K*α radiation
μ = 0.36 mm^−1^
*T* = 120 K
0.5 × 0.23 × 0.15 mm

#### Data collection

Stoe IPDS 2T diffractometer
12920 measured reflections
4973 independent reflections
4065 reflections with *I* > 2σ(*I*)
*R*_int_ = 0.088

#### Refinement

*R*[*F* ^2^ > 2σ(*F* ^2^)] = 0.054
*wR*(*F* ^2^) = 0.113
*S* = 1.13
4973 reflections
218 parameters
H-atom parameters constrained
Δρ_max_ = 0.29 e Å^−3^
Δρ_min_ = −0.34 e Å^−3^
Absolute structure: Flack (1983 ▶), 2099 Friedel pairs
Flack parameter: −0.11 (7)

### 

Data collection: *X-AREA* (Stoe & Cie, 2005 ▶); cell refinement: *X-AREA*; data reduction: *X-RED32* (Stoe & Cie, 2005 ▶); program(s) used to solve structure: *SHELXS97* (Sheldrick, 2008 ▶); program(s) used to refine structure: *SHELXL97* (Sheldrick, 2008 ▶); molecular graphics: *ORTEP-3 for Windows* (Farrugia, 1997 ▶); software used to prepare material for publication: *WinGX* (Farrugia, 1999 ▶).

## Supplementary Material

Crystal structure: contains datablock(s) I, global. DOI: 10.1107/S1600536812000724/bt5777sup1.cif

Structure factors: contains datablock(s) I. DOI: 10.1107/S1600536812000724/bt5777Isup2.hkl

Supplementary material file. DOI: 10.1107/S1600536812000724/bt5777Isup3.cml

Additional supplementary materials:  crystallographic information; 3D view; checkCIF report

## Figures and Tables

**Table 1:** Hydrogen-bond geometry (Å, °)

*D*—H⋯*A*	*D*—H	H⋯*A*	*D*⋯*A*	*D*—H⋯*A*
C3—H3*A*⋯Cl1^i^	0.97	2.81	3.525 (3)	131

Symmetry code: (i) 
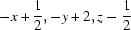
.

**Fig. 1.**
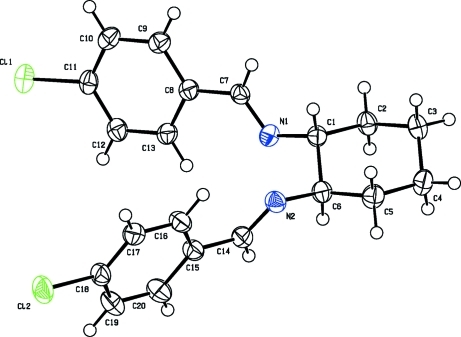
The molecular structure of the title compound with displacement ellipsoids drawn at 50% probability level.

**Fig. 2.**
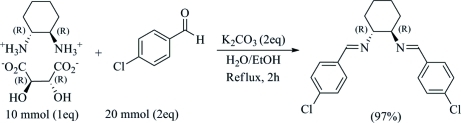
The reaction scheme for the synthesis of the title compound.

**Fig. 3.**
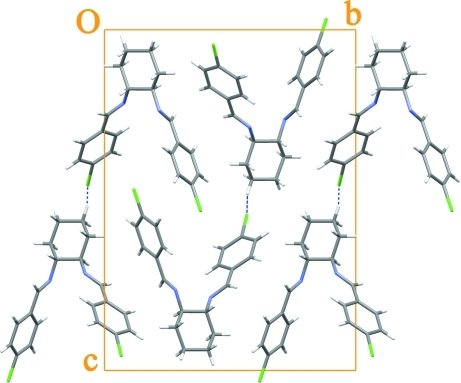
The intermolecular C—H···Cl hydrogen bonds are shown as blue dashed lines.

## supplementary crystallographic information

### Comment

The class of chiral chelating Schiff bases are significant compounds in chemistry so that several reviews have been published on these substances (Da Silva *et al.*, 2011; Przybylski *et al.*, 2009; Gupta and Sutar 2008). Because of their stereochemical structures as well as their industrial properties (Dhar & Taploo, 1982) and potent biological activities (Da Silva *et al.*, 2011; Przybylski *et al.*, 2009) they are very attractive synthetic targets. Furthermore, it should be stressed that these useful and recyclable materials have been widely used in various enantioselective reactions, such as cyclopropanation (Munslow *et al.*, 2001), aziridination (Gillespie *et al.*, 2002), epoxidation (Kureshy *et al.*, 2001), Diels-Alder reaction (Takenaka *et al.*, 2002) as ligands or catalysts.

The asymmetric unit of the title compound which contains one molecule of related Schiff base compound is shown in Fig. 1. The reaction scheme for the synthesis of the title Schiff base is presented in Fig. 2. The bond distances and angles in the title compound are in agreement with related structures (Fan *et al.*, 2011; Glidewell *et al.*, 2005; Saleh Salga *et al.*, 2010). The crystal structure is stabilized by an intermolecular C—H···Cl hydrogen bond (Fig. 3 & Table 1).

### Experimental

In a 25 ml two-necked round bottom flask with a reflux condenser, (*R*,*R*)-1,2-diammoniumcyclohexane mono-(+)-tartrate (2.64 g 10 mmol, 2 eq) and K_2_CO_3_ (2.76 g 20 mmol, 2 eq) were dissolved in H_2_O (3 ml) (Larrow & Jacobsen 1998). The mixture was stirred and heated gently (~50 °C) for 10 min. Then a solution of *p*-chlorobenzaldehyde (2.8 g 20 mmol, 2 eq) in EtOH (10 ml) was poured in dropping funnel and added dropwise. The reaction mixture was stirred and refluxed for further 2 hrs. The mixture was cooled to room temperature and concentrated *in vacuo*. The residue was dissolved in CH_2_Cl_2_ (10 ml) and washed with saturated sodium bicarbonate (5 ml) and dried over Na_2_SO_4_. The organic layer was evaporated to yield crude product. Recrystallization in hot EtOH (7 ml) afford desired compound as colorless needles. 3.47 g, 97% yield, mp. 150 °C (mp 148–150°C), [α]^20^_D_= -308° (c=1, CHCl_3_) ([α]^20^_D_= -136 (*c*=1, CHCl_3_))(Periasamy *et al.*, 2001).

### Refinement

All hydrogen atoms were positioned geometrically and refined as riding atoms with C—H = 0.93–0.98 Å and *U*iso(H) = 1.2 *U*eq(C).

### Crystal data

**Table:**

C_20_H_20_Cl_2_N_2_	*F*(000) = 752
*M**_r_* = 359.28	*D*_x_ = 1.293 Mg m^−^^3^
Orthorhombic, *P*2_1_2_1_2_1_	Mo *K*α radiation, λ = 0.71073 Å
Hall symbol: P 2ac 2ab	Cell parameters from 4973 reflections
*a* = 5.5058 (11) Å	θ = 2.3–29.2°
*b* = 15.734 (3) Å	µ = 0.36 mm^−^^1^
*c* = 21.302 (4) Å	*T* = 120 K
*V* = 1845.4 (6) Å^3^	Needle, colorless
*Z* = 4	0.5 × 0.23 × 0.15 mm

### Data collection

**Table:**

Stoe IPDS 2T diffractometer	4065 reflections with *I* > 2σ(*I*)
Radiation source: fine-focus sealed tube	*R*_int_ = 0.088
graphite	θ_max_ = 29.2°, θ_min_ = 2.3°
Detector resolution: 0.15 mm pixels mm^-1^	*h* = −7→7
rotation method scans	*k* = −19→21
12920 measured reflections	*l* = −29→29
4973 independent reflections	

### Refinement

**Table:**

Refinement on *F*^2^	Hydrogen site location: inferred from neighbouring sites
Least-squares matrix: full	H-atom parameters constrained
*R*[*F*^2^ > 2σ(*F*^2^)] = 0.054	*w* = 1/[σ^2^(*F*_o_^2^) + (0.0326*P*)^2^ + 0.5925*P*] where *P* = (*F*_o_^2^ + 2*F*_c_^2^)/3
*wR*(*F*^2^) = 0.113	(Δ/σ)_max_ = 0.002
*S* = 1.13	Δρ_max_ = 0.29 e Å^−^^3^
4973 reflections	Δρ_min_ = −0.34 e Å^−^^3^
218 parameters	Extinction correction: *SHELXL97* (Sheldrick, 2008), Fc^*^=kFc[1+0.001xFc^2^λ^3^/sin(2θ)]^-1/4^
0 restraints	Extinction coefficient: 0.0059 (11)
Primary atom site location: structure-invariant direct methods	Absolute structure: Flack (1983), 2099 Friedel pairs
Secondary atom site location: difference Fourier map	Flack parameter: −0.11 (7)

### Special details

**Table:**

Geometry. All e.s.d.'s (except the e.s.d. in the dihedral angle between two l.s. planes) are estimated using the full covariance matrix. The cell e.s.d.'s are taken into account individually in the estimation of e.s.d.'s in distances, angles and torsion angles; correlations between e.s.d.'s in cell parameters are only used when they are defined by crystal symmetry. An approximate (isotropic) treatment of cell e.s.d.'s is used for estimating e.s.d.'s involving l.s. planes.
Refinement. Refinement of *F*^2^ against ALL reflections. The weighted *R*-factor *wR* and goodness of fit *S* are based on *F*^2^, conventional *R*-factors *R* are based on *F*, with *F* set to zero for negative *F*^2^. The threshold expression of *F*^2^ > σ(*F*^2^) is used only for calculating *R*-factors(gt) *etc*. and is not relevant to the choice of reflections for refinement. *R*-factors based on *F*^2^ are statistically about twice as large as those based on *F*, and *R*- factors based on ALL data will be even larger.

### Fractional atomic coordinates and isotropic or equivalent isotropic displacement parameters (Å^2^)

**Table:**

	*x*	*y*	*z*	*U*_iso_*/*U*_eq_	
Cl1	−0.02567 (12)	1.06658 (5)	0.95529 (3)	0.03732 (17)	
Cl2	1.02450 (16)	0.61772 (5)	1.03022 (3)	0.0446 (2)	
C9	0.5215 (5)	1.08153 (16)	0.83309 (11)	0.0302 (5)	
H9	0.6498	1.1182	0.8246	0.036*	
N2	0.9823 (4)	0.78245 (13)	0.73687 (9)	0.0292 (5)	
C3	1.0135 (5)	0.91257 (17)	0.56137 (10)	0.0316 (5)	
H3A	0.9729	0.9310	0.5192	0.038*	
H3B	1.1598	0.9422	0.5742	0.038*	
C18	0.9686 (5)	0.65212 (16)	0.95383 (11)	0.0333 (6)	
N1	0.6607 (4)	0.92267 (14)	0.71446 (9)	0.0284 (5)	
C12	0.1355 (5)	0.97300 (18)	0.85723 (11)	0.0293 (5)	
H12	0.0050	0.9371	0.8655	0.035*	
C8	0.4930 (5)	1.00840 (15)	0.79711 (10)	0.0258 (5)	
C10	0.3615 (5)	1.10062 (18)	0.88148 (12)	0.0319 (6)	
H10	0.3825	1.1493	0.9057	0.038*	
C7	0.6765 (5)	0.98715 (16)	0.74955 (10)	0.0256 (5)	
H7	0.8102	1.0228	0.7451	0.031*	
C14	0.8364 (5)	0.73657 (17)	0.76733 (12)	0.0302 (6)	
H14	0.6916	0.7206	0.7482	0.036*	
C11	0.1706 (5)	1.04590 (17)	0.89291 (11)	0.0279 (5)	
C1	0.8652 (5)	0.90495 (16)	0.67300 (11)	0.0269 (5)	
H1	1.0099	0.9347	0.6884	0.032*	
C15	0.8846 (5)	0.70693 (17)	0.83196 (12)	0.0301 (6)	
C2	0.8063 (5)	0.93512 (19)	0.60586 (11)	0.0301 (5)	
H2A	0.6575	0.9083	0.5916	0.036*	
H2B	0.7814	0.9962	0.6058	0.036*	
C17	1.1362 (6)	0.70438 (18)	0.92486 (12)	0.0339 (6)	
H17	1.2761	0.7210	0.9460	0.041*	
C13	0.2957 (5)	0.95433 (17)	0.80948 (11)	0.0285 (6)	
H13	0.2732	0.9057	0.7854	0.034*	
C4	1.0598 (6)	0.81726 (19)	0.56146 (11)	0.0366 (6)	
H4A	1.1963	0.8045	0.5343	0.044*	
H4B	0.9183	0.7880	0.5451	0.044*	
C5	1.1139 (6)	0.78524 (18)	0.62769 (12)	0.0349 (6)	
H5A	1.1323	0.7239	0.6268	0.042*	
H5B	1.2658	0.8096	0.6421	0.042*	
C6	0.9115 (5)	0.80889 (17)	0.67350 (11)	0.0292 (6)	
H6	0.7621	0.7792	0.6615	0.035*	
C19	0.7602 (5)	0.6264 (2)	0.92339 (13)	0.0382 (7)	
H19	0.6486	0.5912	0.9433	0.046*	
C16	1.0941 (5)	0.73191 (17)	0.86384 (12)	0.0328 (6)	
H16	1.2062	0.7672	0.8441	0.039*	
C20	0.7205 (5)	0.65407 (19)	0.86231 (13)	0.0372 (6)	
H20	0.5809	0.6368	0.8413	0.045*	

### Atomic displacement parameters (Å^2^)

**Table:**

	*U*^11^	*U*^22^	*U*^33^	*U*^12^	*U*^13^	*U*^23^
Cl1	0.0353 (3)	0.0513 (4)	0.0254 (2)	0.0067 (3)	0.0022 (3)	−0.0030 (3)
Cl2	0.0633 (5)	0.0419 (4)	0.0286 (3)	0.0112 (4)	0.0068 (3)	0.0108 (3)
C9	0.0313 (13)	0.0296 (12)	0.0298 (10)	−0.0005 (12)	−0.0019 (11)	−0.0020 (10)
N2	0.0323 (11)	0.0293 (10)	0.0260 (9)	−0.0006 (10)	−0.0026 (10)	0.0040 (8)
C3	0.0306 (13)	0.0398 (14)	0.0244 (10)	0.0026 (12)	0.0017 (11)	0.0063 (10)
C18	0.0463 (15)	0.0281 (12)	0.0257 (10)	0.0069 (13)	0.0033 (12)	0.0060 (10)
N1	0.0292 (11)	0.0311 (11)	0.0248 (9)	0.0003 (10)	−0.0013 (8)	−0.0026 (9)
C12	0.0284 (13)	0.0353 (14)	0.0242 (11)	−0.0005 (12)	−0.0041 (10)	0.0025 (11)
C8	0.0280 (13)	0.0271 (12)	0.0221 (9)	0.0014 (11)	−0.0050 (10)	0.0011 (8)
C10	0.0356 (14)	0.0317 (14)	0.0283 (11)	0.0033 (12)	−0.0057 (11)	−0.0058 (10)
C7	0.0286 (12)	0.0261 (12)	0.0223 (10)	−0.0007 (10)	−0.0018 (10)	0.0039 (10)
C14	0.0313 (14)	0.0307 (14)	0.0287 (12)	−0.0028 (12)	0.0007 (11)	0.0002 (11)
C11	0.0291 (12)	0.0340 (14)	0.0206 (10)	0.0089 (11)	−0.0021 (10)	0.0017 (10)
C1	0.0267 (12)	0.0299 (13)	0.0240 (10)	−0.0016 (11)	0.0006 (10)	−0.0006 (10)
C15	0.0341 (13)	0.0268 (13)	0.0295 (12)	0.0005 (11)	0.0025 (11)	0.0038 (10)
C2	0.0307 (12)	0.0337 (14)	0.0259 (11)	0.0052 (12)	0.0000 (10)	0.0050 (11)
C17	0.0394 (15)	0.0322 (14)	0.0301 (13)	−0.0022 (13)	−0.0025 (11)	0.0001 (11)
C13	0.0346 (13)	0.0285 (13)	0.0225 (10)	−0.0002 (11)	−0.0058 (10)	0.0001 (10)
C4	0.0426 (16)	0.0417 (15)	0.0253 (11)	0.0037 (14)	0.0015 (11)	−0.0037 (11)
C5	0.0404 (16)	0.0330 (14)	0.0312 (13)	0.0088 (13)	0.0005 (12)	0.0006 (11)
C6	0.0341 (14)	0.0299 (13)	0.0235 (11)	0.0002 (12)	−0.0031 (10)	0.0020 (10)
C19	0.0355 (15)	0.0385 (16)	0.0405 (14)	−0.0014 (13)	0.0086 (12)	0.0122 (13)
C16	0.0362 (15)	0.0300 (14)	0.0323 (13)	−0.0047 (12)	0.0008 (11)	0.0062 (11)
C20	0.0313 (14)	0.0388 (16)	0.0415 (15)	−0.0053 (13)	0.0012 (12)	0.0060 (13)

### Geometric parameters (Å, °)

**Table:**

Cl1—C11	1.743 (3)	C14—C15	1.478 (4)
Cl2—C18	1.742 (2)	C14—H14	0.9300
C9—C10	1.389 (4)	C1—C6	1.533 (4)
C9—C8	1.391 (3)	C1—C2	1.541 (3)
C9—H9	0.9300	C1—H1	0.9800
N2—C14	1.260 (3)	C15—C20	1.388 (4)
N2—C6	1.466 (3)	C15—C16	1.395 (4)
C3—C4	1.521 (4)	C2—H2A	0.9700
C3—C2	1.525 (3)	C2—H2B	0.9700
C3—H3A	0.9700	C17—C16	1.390 (3)
C3—H3B	0.9700	C17—H17	0.9300
C18—C19	1.379 (4)	C13—H13	0.9300
C18—C17	1.382 (4)	C4—C5	1.527 (4)
N1—C7	1.263 (3)	C4—H4A	0.9700
N1—C1	1.458 (3)	C4—H4B	0.9700
C12—C13	1.378 (4)	C5—C6	1.527 (4)
C12—C11	1.389 (4)	C5—H5A	0.9700
C12—H12	0.9300	C5—H5B	0.9700
C8—C13	1.404 (4)	C6—H6	0.9800
C8—C7	1.469 (3)	C19—C20	1.390 (4)
C10—C11	1.380 (4)	C19—H19	0.9300
C10—H10	0.9300	C16—H16	0.9300
C7—H7	0.9300	C20—H20	0.9300
			
C10—C9—C8	121.1 (3)	C16—C15—C14	120.9 (2)
C10—C9—H9	119.5	C3—C2—C1	110.3 (2)
C8—C9—H9	119.5	C3—C2—H2A	109.6
C14—N2—C6	117.9 (2)	C1—C2—H2A	109.6
C4—C3—C2	110.7 (2)	C3—C2—H2B	109.6
C4—C3—H3A	109.5	C1—C2—H2B	109.6
C2—C3—H3A	109.5	H2A—C2—H2B	108.1
C4—C3—H3B	109.5	C18—C17—C16	119.5 (3)
C2—C3—H3B	109.5	C18—C17—H17	120.3
H3A—C3—H3B	108.1	C16—C17—H17	120.3
C19—C18—C17	121.4 (2)	C12—C13—C8	120.3 (2)
C19—C18—Cl2	119.7 (2)	C12—C13—H13	119.9
C17—C18—Cl2	119.0 (2)	C8—C13—H13	119.9
C7—N1—C1	117.3 (2)	C3—C4—C5	111.0 (2)
C13—C12—C11	119.4 (3)	C3—C4—H4A	109.4
C13—C12—H12	120.3	C5—C4—H4A	109.4
C11—C12—H12	120.3	C3—C4—H4B	109.4
C9—C8—C13	119.0 (2)	C5—C4—H4B	109.4
C9—C8—C7	119.4 (2)	H4A—C4—H4B	108.0
C13—C8—C7	121.5 (2)	C6—C5—C4	111.6 (2)
C11—C10—C9	118.6 (2)	C6—C5—H5A	109.3
C11—C10—H10	120.7	C4—C5—H5A	109.3
C9—C10—H10	120.7	C6—C5—H5B	109.3
N1—C7—C8	122.9 (2)	C4—C5—H5B	109.3
N1—C7—H7	118.5	H5A—C5—H5B	108.0
C8—C7—H7	118.5	N2—C6—C5	109.0 (2)
N2—C14—C15	123.1 (3)	N2—C6—C1	109.3 (2)
N2—C14—H14	118.4	C5—C6—C1	110.9 (2)
C15—C14—H14	118.4	N2—C6—H6	109.2
C10—C11—C12	121.6 (2)	C5—C6—H6	109.2
C10—C11—Cl1	119.3 (2)	C1—C6—H6	109.2
C12—C11—Cl1	119.0 (2)	C18—C19—C20	118.6 (3)
N1—C1—C6	108.2 (2)	C18—C19—H19	120.7
N1—C1—C2	109.9 (2)	C20—C19—H19	120.7
C6—C1—C2	110.2 (2)	C17—C16—C15	120.4 (3)
N1—C1—H1	109.5	C17—C16—H16	119.8
C6—C1—H1	109.5	C15—C16—H16	119.8
C2—C1—H1	109.5	C15—C20—C19	121.5 (3)
C20—C15—C16	118.7 (2)	C15—C20—H20	119.3
C20—C15—C14	120.4 (3)	C19—C20—H20	119.3
			
C10—C9—C8—C13	1.3 (4)	C9—C8—C13—C12	−1.0 (4)
C10—C9—C8—C7	−175.4 (2)	C7—C8—C13—C12	175.6 (2)
C8—C9—C10—C11	−0.7 (4)	C2—C3—C4—C5	−56.8 (3)
C1—N1—C7—C8	−174.9 (2)	C3—C4—C5—C6	55.3 (3)
C9—C8—C7—N1	−178.8 (2)	C14—N2—C6—C5	−127.0 (3)
C13—C8—C7—N1	4.6 (4)	C14—N2—C6—C1	111.7 (3)
C6—N2—C14—C15	−178.9 (2)	C4—C5—C6—N2	−175.6 (2)
C9—C10—C11—C12	−0.2 (4)	C4—C5—C6—C1	−55.2 (3)
C9—C10—C11—Cl1	177.6 (2)	N1—C1—C6—N2	−63.4 (3)
C13—C12—C11—C10	0.5 (4)	C2—C1—C6—N2	176.4 (2)
C13—C12—C11—Cl1	−177.32 (19)	N1—C1—C6—C5	176.4 (2)
C7—N1—C1—C6	138.6 (2)	C2—C1—C6—C5	56.2 (3)
C7—N1—C1—C2	−101.0 (3)	C17—C18—C19—C20	0.0 (4)
N2—C14—C15—C20	−177.8 (3)	Cl2—C18—C19—C20	−179.5 (2)
N2—C14—C15—C16	2.7 (4)	C18—C17—C16—C15	−0.1 (4)
C4—C3—C2—C1	58.1 (3)	C20—C15—C16—C17	−0.2 (4)
N1—C1—C2—C3	−176.9 (2)	C14—C15—C16—C17	179.3 (3)
C6—C1—C2—C3	−57.8 (3)	C16—C15—C20—C19	0.4 (4)
C19—C18—C17—C16	0.2 (4)	C14—C15—C20—C19	−179.1 (3)
Cl2—C18—C17—C16	179.7 (2)	C18—C19—C20—C15	−0.3 (4)
C11—C12—C13—C8	0.1 (4)		

### Hydrogen-bond geometry (Å, °)

**Table:**

*D*—H···*A*	*D*—H	H···*A*	*D*···*A*	*D*—H···*A*
C3—H3A···Cl1^i^	0.97	2.81	3.525 (3)	131.

Symmetry codes: (i) −*x*+1/2, −*y*+2, *z*−1/2.
